# Medicinal Plants in Poultry During Post‐Antibiotic Era in One‐Health Approach: An Extensive Review

**DOI:** 10.1002/vms3.71119

**Published:** 2026-08-28

**Authors:** Alireza Talebi, Arman Savari

**Affiliations:** ^1^ Department of Poultry Health and Diseases Faculty of Veterinary Medicine Urmia University Urmia Iran

**Keywords:** bacterial resistance, medicinal plants, one‐health, post‐antibiotic era, poultry

## Abstract

**Background:**

Increasing drug resistance, together with possible transmission of these phenomena to other pathogenic agents, as well as the dissemination of resistant microbes and drug residues to the environment, poses existing challenges for human health. The journey in history of medicine indicates that the post‐antibiotic era has been initiated and the application of medicinal plants is a promising alternative to antibiotics.

**Objectives:**

The aim of this review was to have a glance at available medicinal plant products in modern poultry production in the post‐antibiotic era. However, the globalization of one‐health needs efforts from both scientists and policy makers to work firmly in a harmonized mission continuously, and all nations must be simultaneously involved in this process to fulfil their obligation properly.

**Methods:**

This review is focused on the role of the well‐known medicinal plants in poultry and method of reviewing includes a brief introduction, history, the present status and prospective future by critical reviewing the published literatures.

**Results:**

An extensive application of natural medicine in poultry during the post‐antibiotic era in a comprehensive holistic one‐health was reviewed, and some of the most relevant commercially available products were also mentioned as a footprint to encourage the researchers to include all the necessary parameters for practical application of the medicinal plant products during their study in details. Some recommendations/future directions on achieving the goals and completing the mission were also proposed.

**Conclusion:**

Application of the medicinal plants in intensive poultry industry is the best solution for production of antibiotic‐free foods during the post‐antibiotic era.

## Highlights

- The Earth is home for all‐living creatures
- Human being has obligation to save our living planet for everyone via one‐health mission
- Natural medicine is the ideal alternatives for antibiotics
- Role of avian medicine in one‐health

## Graphical abstract

One‐Health is a gift of 21st century and human being must fulfill his obligations. Poultry industry is growing steadily and faster than other food animals' industries, and intensive poultry production is the frontier in producing antibiotic‐free foods for human beings. Post‐antibiotic era has already been initiated and scientific natural medication is the best alternative for antibiotics in a One‐Health approach.

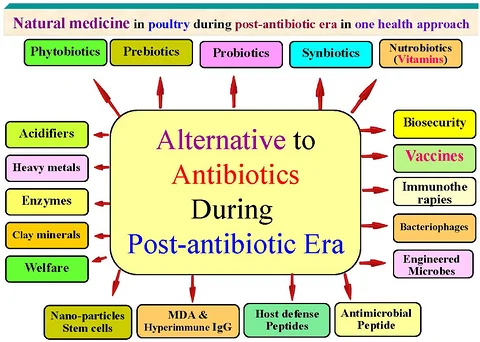

## 1 Introduction

Since ancient times, human beings rely heavily on the surrounding environment to meet the needs, such as for food, shelter, clothing and medicines (Haniarti et al. 2019), and the poultry industry is contributing significantly to the food industry. Unfortunately, the worldwide excess usage of antimicrobial drugs for growth promotion, prevention and treatment of poultry diseases have led to the emerging of resistant avian pathogens to available antibiotics (Hartady et al. 2021; Mohammadzadeh et al. 2024; Singh et al. 2024; Zhou et al. 2020) and increasing antimicrobial resistance (AMR) (Hannan et al. 2024) as well as multidrug‐resistant (MDR) bacterial strains (Abreu et al. 2023) are a major health threat worldwide (Naghavi et al. 2022). Regarding the magnitude of drug‐resistance challenge, 1.27 million deaths were attributed to drug‐resistant infections in 2019 (Patel et al. 2023), and it has been estimated that 8.22 million deaths associated with AMR could occur globally in 2050 (Murray et al. 2024). Therefore, antibiotic resistance is a significant risk for the global health and necessitates promoting a one‐health holistic approach based on Food and Agriculture Organization (FAO), World Organization for Animal Health (OIE) and World Health Organization (WHO) guidelines to combat the challenge (Aslam et al. 2021). Holstering national strategy and action plans for AMR governance is required to overcome AMR at the national, regional and global levels (Patel et al. 2023). Although, restriction on the usage of antibiotic growth promoters as feed‐additive are increasing (Hajati et al. 2014), however, there is no insight discovery of new synthetic antimicrobials at least for the time being (Hou et al. 2023). However, available data indicate that the medicinal plant products have antimicrobial properties and could be the best alternative for AMR drugs with a promising solution for the post‐antibiotic era (Abdallah et al. 2023; Orimaye et al. 2024; Sugiharto 2021) in tackling infections in both poultry and human health (Ivanova et al. 2024; Pashaei et al. 2024; Rastogi et al. 2015; Romero et al. 2022; Sohani 2021). Avian medicine is considered a preventive medicine and there is no doubt that predictable diseases can be prevented, and poultry veterinarians should believe that the prevention of poultry diseases is preferred to their treatment in an intervention strategy protocol. More interesting, the bioactive compounds of most medicinal plants act as a preventive medicine, and their use fit into all existing prevention strategies (Garg et al. 2021; Sofowora et al. 2013; Sohani 2021). The aim of this review was to have a glance at available medicinal plant products in modern poultry production and to encourage the incoming researchers to be objective‐oriented and focus on the neglected objectives of the specific subjects of the past to address the present and future challenges.

## 2 Method of Review

### 2.1 Research Design

Method of reviewing the topics (post‐antibiotic era, one‐health, medicinal plants and poultry) of this research includes a brief introduction, history and the present status and prospective future by critical reviewing the published literatures.

### 2.2 Search Strategy and Selection Criteria

This extensive review was intended to remind the remarkable present/future challenges of the poultry industry including the most important viral diseases (AI, AIB, IBD and ND), bacterial infections (colibacillosis, mycoplasmosis and salmonellosis), parasitic manifestation (coccidiosis) and fungal infections (Armor 2020; Arné and Lee 2020; Cervantes et al. 2020; Eterradossi and Saif 2020; Gast and Porter 2020; Jackwood and de Wit 2020; Miller and Koch 2020; Nolan et al. 2020; Swayne et al. 2020; Yehia et al. 2023) in poultry production to orient the incoming researches and to encourage to commercialize products of the recognized medicinal plants with a promising preventive/treatment activities.

### 2.3 Post‐Antibiotic Era

The history of medicine may have three phases, including pre‐antibiotic era (up to 1935), antibiotic era (1935‐continue) and post‐antibiotic era (initiated in 2010). Undoubtedly, a few decades of the antibiotic era were a golden age to treat the challenging diseases (Abdallah et al. 2023); however, extensive use of antibiotics in food animal production constitutes a major contributing factor to the current AMR crisis (Van et al. 2020). However, limitation on discovery of new drugs, increasing resistance of pathogenic agents to available drugs (Abreu et al. 2023; Alanis 2005; Qian et al. 2022; Morrow 2024), emergence of multidrug resistance (Islam et al. 2024) and considerable variation in AMR governance strategies between countries (Patel et al. 2023) had a very adverse impacts on application of antibiotics worldwide. Meanwhile, transmission of resistance phenomena vertically and horizontally from drug‐resistant microbes to other pathogenic agents (Alanis 2005; Hedman et al. 2020) and drug residues in foods (Diaz‐Sanchez et al. 2015), together with an increasing demand for antibiotic‐free products, have become a major global healthcare problem in the 21st century. On the other hand, medicinal plants are a potential source to develop novel therapeutic medicine (Abbas et al. 2022) and improve the safety of organic poultry products for human consumption (Abd El‐Hack et al. 2022).

### 2.4 One‐Health

Increasing human population and detrimental environmental activities have led to pollution, depletion of natural resources and climate change (Abdallah et al. 2023). On the other hand, contribution of antimicrobial usage from animals and plants to the overall burden of AMR threating of both animals, plants and human health (Naghavi et al. 2022; Roth et al. 2019) and leading to a genuine understanding one‐health in order to address the global food safety challenges and natural disasters (climate change, global warming, atmospheric phenomena including hurricanes, floods, wildfires, prolonged drought and air pollution) (Abreu et al. 2023; Bolan et al. 2024; Qian et al. 2022; Gomes et al. 2023; Hoffmann et al. 2022). Nowadays, regardless of the geographical, social, skin colour and language differences, health for all is the best way to keep the human family safe. Concerning animal health, it should be emphasized that animals are the main sources of our food, clothes and shoes; without animal health, our food could be in danger. Moreover, plant health is also more important than animal health because plants not only are the main sources (80%) of food for animals and human beings, but also provide 98% of the oxygen we breathe. In reality, plants by producing O_2_ and reducing CO_2_, act as a big air‐refinery factory and play not only a vital role in human and animal life but also in climate safety too. However, despite the high benefits of plants, zoonotic/opportunistic phytopathogens should not be neglected (Kim et al. 2020). Concerning environmental health, human beings have to acknowledge that planet Earth is home to all, and we have to believe in a one‐health holistic approach, which is a gift of the 21st century, starting in 2010. There is no doubt that the one‐health approach is a vital framework strategy involving policymakers, healthcare professionals, veterinarians, farmers, environmentalists and the whole public (Endale et al. 2023). Regarding the poultry industry, the use of effective strategies can help prevent occupational exposure and protect the health of workers, consumers, animals and environment (Gomes et al. 2023). Veterinarians play a pivotal role in advancing one‐health (Lutful Kabir 2025).

### 2.5 Poultry Industry

**FIGURE 1:**
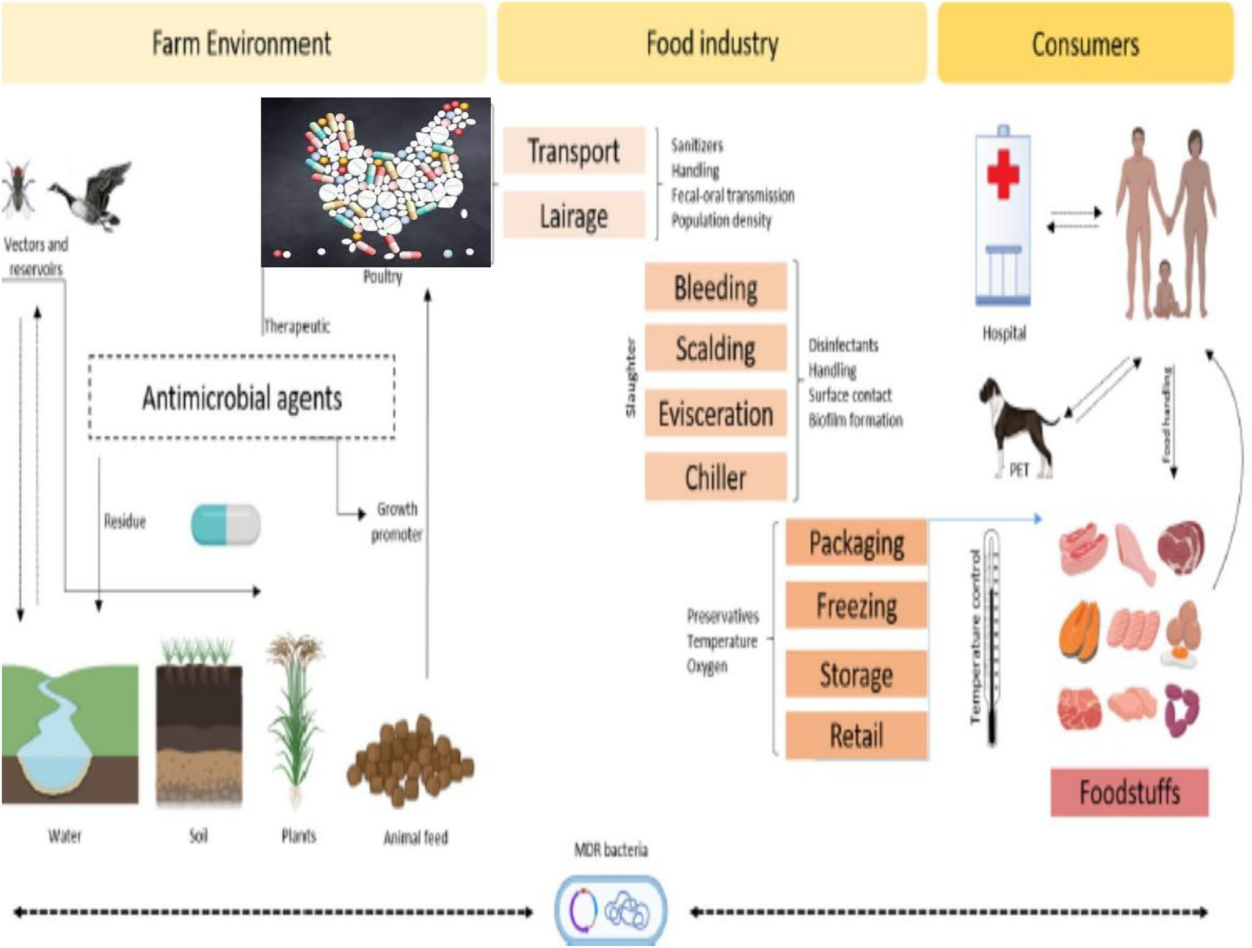
Drugs residues and potential transmission routes of antimicrobial‐resistant bacteria/antimicrobial resistance genes transmission are the primary concern of public health (de Mesquita Souza Saraiva et al. 2022).

Poultry have been on the Earth for over 150 million years since the Jurassic era, and modern birds include more than 10,000 species distributed worldwide (Brusatte et al. 2015). Nowadays, poultry production is a vital component of modern agriculture, and poultry products are the main affordable, valuable sources of protein (Farrell 2013). There is a prediction that demand for poultry meat per capita consumption will increase sharply in the near future (Hedman et al. 2020), and poultry products are predicted to quadruple globally by 2050 (Attia et al. 2024). Therefore, it is necessary to sustain the poultry industry in the absence of synthetic growth promoters in the poultry diets (Abdel‐Moneim et al. 2024). Nowadays, the expansion of AMR, limitations in the discovery of new antibiotics and the presence of drug residue in poultry products, together with growing demands for antibiotic‐free foods, are multiple challenges at present and in the near future (Korver et al. 2023). On the other hand, excessive/inappropriate/misuse of drugs in poultry farms leads to drug resistance, drug dissemination into the environment and drug residues in products, besides food‐borne diseases (Hedman et al. 2020; Patyra et al. 2020; Ventura da Silva 2013). Unfortunately, AMR in poultry pathogens (Nhung et al. 2017) and drug residues in poultry eggs and meat (Figure 1) (Mund et al. 2016; Patel et al. 2018; Pratiwi et al. 2023; Sani et al. 2023), as well as in poultry manure (Lacroix et al. 2023; Li et al. 2023a; Pokrant et al. 2021; Rashid et al. 2023), are increasing over time. Although poultry manure is a good fertilizer (Dobbins 2023; Manogaran et al. 2022; Zhang et al. 2017), it contains pathogenic/harmful resistant microorganisms disseminated into environment (Pokrant et al. 2021; Qian et al. 2022) causing soil and water contamination (Bolan et al. 2024; Lacroix et al. 2023; Patyra et al. 2020). Recent studies revealed that AMR genes, which are disseminated from human and animal waste to the environment, remain unaltered for many years (Van et al. 2020) and affect human health via transmission by soil microbiome (Banerjee and van der Heijden 2023; Qian et al. 2022). In poultry production, 90% of consumed doses of administered drugs may be found in the manure, and drug dissemination evolves in environmental pollution (Oubouyahia et al. 2025; Patyra et al. 2020). Therefore, application of herbal nutraceuticals/herbal medicine as an alternative intervention strategy is necessary in the post‐antibiotic era (Abdel‐Moneim et al. 2024; El‐Sabrout et al. 2023). However, the herbal products can be supplemented to the poultry diet individually or in combination to improve performance (El‐Sabrout et al. 2023), and the medicinal plants can be useful for enrichment of poultry products (Campbell et al. 2019; El‐Sabrout et al. 2023; Farrell 2013).

### 2.6 Medicinal Plants in Poultry Production

**FIGURE 2:**
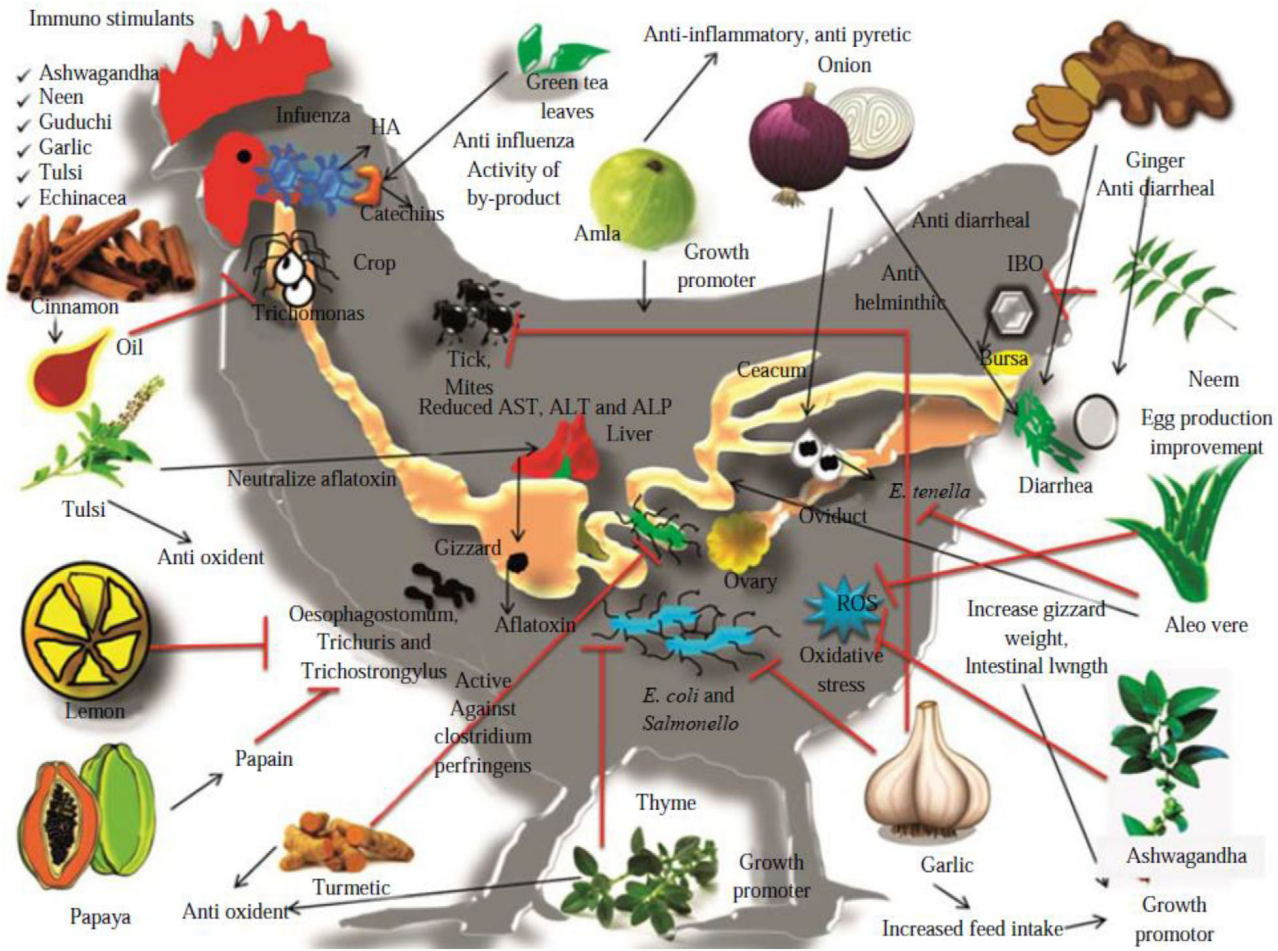
An overview of medicinal plants and their beneficial effects in poultry (Dhama et al. 2015).

The utilization of medicinal plants is as old as human civilization, and there are over 300,000 species of plants (Muthamilselvan et al. 2016); however, a few percentage of them have been used as raw materials for traditional medicine (Haniarti et al. 2019). Recently, some medicinal plants comprising of 9225 phytochemicals have been provided in an accessible database (Kiewhuo et al. 2022) and the most important medicinal plants which are common in livestock production are *Acacia angustissima*, *Annona senegalensis*, *Artemisia absinthium*, Asafoetida (*Ferula assa‐foetida*), *Azadirachta indica*, *Boswellia frereana*, *Cajanus cajan*, *Carcia papaya*, *Carduus pycnocephalus*, *Chenopodium album*, *Cortez phellodendri*, *Curcuma longa*, Devil's claw, *Drimia sanguinea*, *Echinaceae purpurea*, *Ecklonia cava*, *Elephantorrhiza elephantine*, *Eryngium billardieri* F. Delaroche, *Ficus thonningii* Blume, Germanders (*Teucrium polium*), *Glycyrrhiza glabra*, *Gypsum fibrosum*, *Harrisonia abyssinica* Oliv., *Herba agastaches*, *Hypoxis hemerocallidea*, Lavender (*Lavandula stoechas*), Liquorice (*G. glabra*), *Morinda citrifolia*, *Moringa oleifera*, *Myrsine africana*, *Origanum vulgare*, *Peltophorum africanum*, *Picrorhiza kurroa*, *Rheum nobile*, Savory (*Satureia hortensis* L.), *Sesbania sesban*, *Solanum incanum* L., *Tillandsia recurvata*, *Trichilia claussenii*, Thyme (*Thymus vulgaris*), *Vitex thomasii* De Wild, *Zingiber officinale* (Hannan 2024; Nazari et al. 2023; Shahrajabian et al. 2021; Shahrajabian and Sun 2024). Some species of plants have also been recently identified by the Food and Agriculture Organization as valuable resources for human life (Ehrenberg et al. 2024). In the last decades, phytotherapy has gained great importance (Romero et al. 2022), and the global market value of medicinal plant products exceeded from $100 billion per annum (Sofowora et al. 2013). The World Health Organization (WHO) reported that 80% of the Earth's population relies on traditional medicine for their primary healthcare needs (Dhama et al. 2015; Jamil et al. 2022; Rastogi et al. 2015; Singh et al. 2016). As shown in Figure 2, application of various medicinal plants in poultry is rapidly growing as poultry feed additives (Abdel‐Moneim et al. 2024; Hajati et al. 2014; Nazari et al. 2023) to promote productivity and performance (Abreu et al. 2023; Diaz‐Sanchez et al. 2015) as preventive/treatment medication of poultry diseases (Abreu et al. 2023; Eftekhari‐Hasan‐Abad and Ghaniei 2023; Ghaniei et al. 2023; Jamil et al. 2022; Hartady et al. 2021; Han et al. 2022; Shami et al. 2024) by promoting innate resistance to infections/improvement of poultry immune system (Abreu et al. 2023; Qian et al. 2022; Haniarti et al. 2019; Hartady et al. 2021), in vaccine production for poultry (Hartady et al. 2021) and for enrichment of poultry products (meat and egg) (Dablool et al. 2024; Ghaly et al. 2023; Johanian et al. 2017; Hajati et al. 2014; Hartady et al. 2021).

In general, medicinal plants are rich with some phytochemical bioactive ingredients having biological activities (Omolere and Alagbe 2020), including antiviral (Olafadehan et al. 2020), antimicrobial (Ghaly et al. 2023; Olafadehan et al. 2020), antioxidant (Alagbe et al. 2019), antifungal (Witkowska et al. 2016) and anti‐inflammatory (Nunes et al. 2020) activities. Although some phytochemical compounds, such as tannin compounds, saponins, flavonoids and alkaloids, are common among most of the medicinal plants (Sabdoningrum et al. 2021), testing of herbal products (Indrayanto 2018; Kartini et al. 2019) before their administration is necessary. Therefore, further research is highly recommended to standardize the application of the herbal medicinal product in poultry (Dhama et al. 2015; Diaz‐Sanchez et al. 2015; Orimaye et al. 2024). The routine usage of medicinal plants in poultry could be as follows.

#### 2.6.1 Phytoaddetives (Phytobiotics = Phytogenic)

In the ascent of antibiotic growth promoters, natural bioactive compounds of botanical origin (herbs, botanicals, essential oils and oleoresins) are generally recognized as safe for improving performance (Johanian et al. 2017; Hafez et al. 2022; Nazari et al. 2023; Talebi et al. 2021; Ürüşan and Bölükbaşı 2017) and meat quality of broiler chickens (Aziz‐Aliabadi et al. 2023; El‐Sabrout et al. 2023; Girsang et al. 2019; Gumus and Gelen 2023; Ivanova et al. 2024; Mudalal et al. 2020). In general, the herbal products are involved in maintaining the optimal intestinal digestive functions and improving the histomorphology of the intestine (Diyantoro and Rochmi 2019; Ningsih et al. 2019; Subhan et al. 2023). In addition, the phytoaddetives are also effective in restoring the intestinal damage and digestive enzyme activity following stress/infections (Rahayu et al. 2020). It has been reported that supplementation of 100–200 ppm of thyme (*T. vulgaris*) in the diet not only improves performance but also decreases abdominal fat of broiler chickens (Zhang et al. 2021). Supplementations of *Nigella sativa* powder (Noviani et al. 2020; Talebi et al. 2021), *C. longa* and *Z. officinale* (1 mL/L) (Maksudi et al. 2020), *Averrhoa bilimbi* fruit filtrate (Mareta et al. 2020), *Piliostigma thonningii* (80 mg/L) (Alagbe et al. 2020) and *Citrus aurantium* (40 mg/L) (Alagbe et al. 2022) also improve blood profiles of poultry. Supplementation of 3.5%–5.2% rosemary (*Rosmarinus officinalis*) improved the performance and quality of both fresh and stored eggs (Kedir et al. 2023), and supplementation of 200 mg/Kg diet with nanoprotected rosemary essential oil (REO) improved broiler chickens' lipid profile, immune‐antioxidant status and the intestinal health (Adil et al. 2024). Meanwhile, rosemary ultrafine powder (RUP) as a feed additive, extended breeder service life at an appropriate level of 1.0 g/kg diet (Li et al. 2024). However, some commercial phytobiotics products (CRINA poultry [25–50 mg/kg diet], EXTRACTTM [100 mg/kg diet], Biomin P.E.P 125 poultry [125 g/1000 kg diet], Activo liquid [0.4 %]), have already been available in the poultry market (Diaz‐Sanchez et al. 2015). Moreover, supplementation of roosters' diets with *Cinnamomum comphora* (5000–10000 ppm), camphor (50 mg/kg) or *Z. officinale* (2.5–5 g/kg) showed a positive impact on the breeder male reproductive performance, fertility and hatchability (Raei et al. 2021; Saied et al. 2018). On the other hand, Yucca extract as a green feed additive (500 mg/kg of diet) improves productive performance and egg quality of laying hens (Mao et al. 2023). Overall, the medicinal plants are used as feed additives, in drinking water, feed supplementation and for sanitation (by fogging or inhalation) (Adaszyńska‐Skwirzyńska and Szczerbińska 2017); however, in some herbal products, the drinking water route is preferred to feed supplementation (Akhsan et al. 2020; Diaz‐Sanchez et al. 2015), although the latter is the most routine route in laying hens. However, for successful treatment of respiratory infections including viral (AI, AIB, ND), bacterial (mycoplasmosis) and fungal (aspergillosis) inhalation delivery methods (aerosol, fine spray, dry powder form, smoke for/fumigation) of medicines may have a less off‐target side‐effects (Amorij et al. 2008; Antonissen and Martel 2018) and further high quality controlled trials are recommended (Pashaei et al. 2024). Some of the phyto‐feed additives are presented in Table 1, and a few commercialized herbal feed additive products, including Bioherbal, Activo‐Liquid (0.4%), Hygen pro, Gimax, Biomin P.E.P 125.

**TABLE 1:** The medicinal plants are used as feed additives in poultry production.

Medicinal plant	Optimum dose	Impacts	Reference
*Aloe vera*	0.75% of feed	Performance and carcass traits of broilers	Sunu and Abdurrahman (2019)
Extract of *Morinda citrifolia* L.	0.12% of diet	Improved the health of liver (as indicated by SGOT, SGPT and relative weight of liver)	Ningsih et al. (2019)
*Aloe vera, Morinda citrifolia* or *ginger*	*Aloe vera* (0.75%), *Morinda citrifolia* (5 g/kg), *ginger* (2%)	Performance	Sulistyoningsih (2015)
*Anethum graveolens* L. (Dill plant)	Feeding 0.4 and 0.6%	Improve performance, Reduce cholesterol and triglycerides, Improve microbial flora	Phillips et al. (2023)
Combination of *Moringa oleifera* leaf powder and garlic powder	1% of feed	Reduced ileal *Enterobacteriaceae* population and abdominal fat content and of broilers	Sugiharto et al. (2018)
*Anredera cordifolia* leaf meal	2% of feed	Improved weight gain and feed conversion of broilers	Widodo et al. (2017)
Turmeric powder	1% of feed	Increased total revenue of broiler farmers	Bando et al. (2020)
Turmeric powder	2% of feed or drinking water	Increased weight and length of intestine of broilers	Akhsan and Akbar (2020)
Turmeric extract	800 mg/kg live body weight	Increased haemoglobin level and reduced abdominal fat content	Sugiharto et al. (2011)
*Curcuma xanthorrhiza* and garlic	Maximum of 2% of diet	Improved growth performance of broilers	Lestari et al. (2020)
*Curcuma longa (turmeric)*	100 mg/kg of diet	Performance	Hafez et al. (2022)
*Nigella sativa* powder	4% of feed	Increased carcass weight of broiler exposed to heat stress	Zulkifli et al. (2018)
*Nigella sativa* powder	144 mg /kg body weight/day	Improved haematological condition (haemoglobin and haematocrit levels) of broilers	Noviani et al. (2020)
Extract of *Zingiber zerumbet* (L.) Smith	0.67% to 1% of feed	Restored the intestinal damage and digestive enzyme activity due to Salmonellosis in broilers	Rahayu et al. (2020)
*Zingiber zerumbet* (L.) Smith extract	1% of feed	Decreased fat content of meat, but had no effect on production performance of broilers	Rahayu et al. (2019)
Extract of *Andrographis paniculata*	0.02% of diet	Increased protein and lowered fat content of broiler meat	Supomo et al. (2016)
*Echinacea purpurea* powder	7.5 g/kg of diet	Increase egg production, egg mass, HDL cholesterol level)	Johanian et al. (2017)
Leaves of rosemary (Rosmarinus officinalis)	2.5–7.5 g/kg of diet	Attenuate age‐related sub‐fertility in senescent roosters	https://poultrydvm.com/supplement/rosemary
	200 mg/kg of diet	Improve lipid profile, immune‐antioxidant status, and intestinal health	Adil et al. (2024)
	0.5% of diet	Increase performance	Wang et al. (2024)
	3.5% and 5.2% of diet	performance and quality of eggs	Kedir et al. (2023)

#### 2.6.2 Antibacterial Activity

Among the avian bacterial pathogens, Salmonella species, *Mycoplasma gallisepticum* and *Escherichia coli* are the most important bacteria in poultry production (Swayne 2020), and because of their vigorous growth, their resistance to existing antibiotics, studies on effective antibacterial herbal medicine against these bacteria have been at the centre of world interest for many decades. Some of the medicinal plants are used as antibacterials, which are presented in Table 2, and the overall antibacterial activity of medicinal plants could be summarized as follows.

**TABLE 2:** The medicinal plants are used as antibacterials in poultry production.

Medicinal plant	Poultry bacterial disease	Optimum dose	Impacts	Reference
Activo liquid	*S. typhimurium* (1.9 × 10^9^) *S. enteritidis* (1.3 × 10^9^)	0.4% in drinking water	Bactericidal activity	https://cealvet.com/natural‐solutions‐against‐salmonella‐in‐poultry
*Psidium guajava* (guava)	*Salmonella typhimorium*	5% concentration In vitro (500–700 µL)	Inhibit the growth	Ibrahim et al. (2011)
*Newbouldia laevis*	*S. Typhimurium*	3.125 mg/ml	Bactericidal activity	Usman and Osuji (2007)
*Psidium guajava* (guava)	*Escherichia coli* (*E. coli*)	5% concentration MIC (200–400 µL)	Inhibit the growth	Ibrahim et al. (2011)
*Curcuma longa* (turmeric)		2 g/Kg of diet		Ürüşan and Bölükbaşı (2017)
		100 mg/kg of diet		Aderemi and Alabi (2023)
*Newbouldia laevis *		MIC = 1.563 mg/kg In vitro test	Inhibit the growth	Usman and Osuji (2007)
*Phyllanthus niruri* Linn	*Mycoplasma gallisepticum*	62.5% In vitro condition	Bactericidal and could eradicate *M. gallisepticum*	Sabdoningrum et al. (2017)

##### 2.6.2.1 Salmonella Species

The most effective medicinal plants and their phytochemical activity against some Salmonella species, including *S. typhymorium* has been reviewed (Almuzaini et al. 2023). Leaves of Eucalyptus (*Eucalyptus globulus* L.) and *Hypericum roeperianum*, fruits of guava (*Psidium guajava*), Tamarind (*Tamarindus indica*) and Thyme (*T. vulgaris*) inhibit the growth of Salmonella spp. (Elisha et al. 2017; Hartady et al. 2021; Ibrahim et al. 2011). Recent studies indicated that a nanoliposome form of *N. sativa* purified oil, together with monophosphorylated lipid A, is a promising antibiotic alternative and immunomodulator to control virulent pandemic drug‐resistant Salmonella pullorum infection in broiler chicks (Ahmad et al. 2025).

##### 2.6.2.2 Mycoplasma Species

The threat of AMR and potential cross‐resistance to disinfectants in *M. gallisepticum* necessitates a comprehensive urgent approach to search for anti‐mycoplasma natural medicines (herbal extracts, essential oils, phytobiotics) (Kamal et al. 2025). The *Phyllanthus niruri* Linn with a concentration of 62.5% is effective on *M. gallisepticum* (MG) in vitro in poultry (Sabdoningrum et al. 2017). Quercetin of Ephedra Houttuynia powder (EHP) is also as effective as Tiamulin in the treatment of MG infections in chickens (Wang et al. 2023), and more interestingly, the efficacy of a Chinese herbal medicinal formula (CHMF) against MG is equal to or better than that of Tiamulin (Ying‐Jie et al. 2022).

##### 2.6.2.3 E. coli

*E. coli* is one of the most important food pathogens, which infects poultry (Nawaz et al. 2024; Watts and Wigley 2024), plants (Islam et al. 2024) and humans (Naghavi et al. 2024). AMR and multidrug resistance in avian pathogenic *E. coli* (APEC) are not only a major concern for poultry health (Hossain et al. 2023; Laopiem et al. 2025; Watts and Wigley 2024) but also pose as a reservoir for extra‐pathogenic *E. coli* (EXPEC), a potential zoonotic threat for human health (Azam et al. 2020; Hu et al. 2022; Nawaz et al. 2024). Unfortunately, there is vertical transmission of APEC through eggs, and consequently, the vertical transmission of antibiotic resistance genes is possible (Joseph et al. 2023). Supplementation of *Salvia rosmarinus* (rosemary), *Allium sativum*, *Allium cepa*, and Mentha spp., and Amla effectively reduces the growth of *E. coli* and its intestinal population (Dalal et al. 2018; Ziarlarimi et al. 2011). Guava (*P. guajava*) extract at a 5% concentration level has a significant inhibitory effect on both *E. coli* O157:H7 serotypes (Ibrahim et al. 2011). A combination of EOs eugenol/carvacrol and eugenol/thymol showed a synergistic effect on *E. coli*, whereby thymol and Carvacrol disintegrated the outer membrane of *E. coli*, making it easier for eugenol to access the bacterial cytoplasm to inhibit their growth (Waliaula et al. 2024).

#### 2.6.3 Antiviral Activity

Among the viral diseases of poultry, avian influenza (AI), infectious bronchitis (IB), Newcastle disease (ND) and infectious bursal disease virus (IBD) are contagious diseases prevalent in different types of poultry production worldwide, and no effective drugs are available for their treatment at present (Swayne 2020). However, some of the medicinal plants have antiviral activity by causing capsid disintegration (Wang et al. 2021; Wani et al. 2021) and stimulating macrophage production, activation of macrophages and stimulating T‐cell‐mediated immune responses (Hartady et al. 2021). It has been reported that Aloe species and Neem leaf extract can be used for the treatment of ND, and combined extracts of Rhizomes *Dryopteridis crassirhizomatis* and *Fructus mume* are effective against IBD (Hartady et al. 2021; Ou et al. 2013). While *Camellia sinensis* is effective for control of AI and inclusion body hepatitis‐hydropericardium (IBH) syndrome, and *Withania somnifera*, *Tinospora cordifolia* and *Asphaltum indica* are able to control chicken anaemia virus (CAV) infection (Latheef et al. 2017; Karimi et al. 2022; Yasmin et al. 2020). A combination of extracts of *Ocimum sanctum*, *W. somnifera*, *Emblica officinalis*, *T. cordifolia* and *A. indica* (shilajit) was effective against AIV H9N2 and IBDV co‐infection (Eladl et al. 2020), and a combination of extracts from *Forsythia suspensa* and *Scutellaria baicalensis* had a synergistic effect on preventing IB in poultry (Feng et al. 2024).

The essential oils of the medicinal plants also have antiviral activities against influenza type A virus (Wani et al. 2021) and avian coronavirus (Okino et al. 2024). Nowadays, essential oils of eucalyptus trees have emerged as an antiviral alternative for the treatment of symptoms of human viruses (Mieres‐Castro et al. 2021). Overall, the antiviral action of medicinal plants suggests that some of the medicinal plants, such as aloe vera (250 µg/mL) may act at multiple stages of life cycle of avian pathogenic viruses (Dutta et al. 2023) and their natural compounds could be a source of new antiviral medicines against avian viral pathogens (Abdelaziz et al. 2024; Ahmadi et al. 2024). Some of the medicinal plants with antiviral activities are presented in Table 3, and a few commercialized antiviral herbal products, including Bronchitis Bio Herbal (Nature's Gold), Oil‐Care and RDCFM, have already been introduced in the poultry market.

**TABLE 3:** The medicinal plants are used as antivirals in poultry production.

Medicinal plant	Poultry viral disease	Optimum dose	Impacts	Reference
*Camellia sinensis* (Green Tee)	Avian influenza (AI)	50 µg/mL in vitro	High inhibitory on replication (reduce viral titer)	Karimi et al. (2022)
		10 g/kg of diet	Reduce replication	Yasmin et al. (2020)
*Oleaceae Fructus Forsythiae* (Phillyrin)		100 µg/mL in vitro	Inhibition of viral RNA polymerase	Y. Chen et al. (2023)
*Dryopteridis crassirhizomatis* *Fructus mume*	Infectious Bursal Disease (IBD)	100–200 mg/kg of body weight	Improve antibody level Reduce virus load in the target organs	Ou et al. (2013)
*Withania somnifera*	Coinfection of AI and IBD	Orga Immu containing a mixture of the herbs. 1 mL/L of drinking water for 5 weeks	Stimulate immune responses to IBD, and relieves the pathogenicity of an AIV H9N2 and IBDV co‐infection	Eladl et al. (2020)
*Tinospora cordifolia*				
*Asphaltum indica*				
*Mangifera indica*				
*Ocimum sanctum*				
*Emblica officinalis*				
*Azadirachta indica* *Commiphora swynnertonii*	Newcastle disease (ND)	4 µg/egg in vitro	Reducing the NDV‐stimulated splenocyte proliferation	Yasmin et al. (2020 Helmy et al. (2007
*Hypericum perforatum* extract (St. John's Wort)	Avian Infectious bronchitis (AIB)	120 mg/kgbw Oral 2‐times per day for 5 days	Antiviral activity (in vitro and in vivo) by reduced mRNA expression levels	H. Chen et al. (2019)
*Forsythia suspensa* extract		80 mg/kg/day of diet	Antiviral activity (prevention & treatment)	Wang et al. (2021)
*Forsythia suspensa* and *Scutellaria baicalensis* extracts Mixture of *Cinnamomi cortex* (Cinnamaldehyde) + Glycerol monolaurate		24 mg/mL + 45 mg/mL (drinking water) for 7 days	Reduction in viral copies Increase of antiviral protein (G3BP1) level in tracheas. Increasing anti‐IBV antibody levels in serum	Feng et al. (2024)
		Prevention: 1.5 mL/L Treatment:1 mL/L in drinking water for 5 days	Reduction of IBV mRNA expression levels Increase IBV‑specific antibody titers	Y. Zhang et al. (2022)
*Withania somnifera*	Chicken anaemia virus (CAV)	Extract 1%	Reduced virus load in Thymus, spleen and liver	Latheef et al. (2017)
*Tinospora cordifolia*		Extract 1%		
*Asphaltum indica*		Extract 0.2%		
*Camellia sinensis* (Green Tea) extract	Inclusion Body Hepatitis (IBH)	100 mg/mL drinking water for 6 days	Reduce mortality rate by 90% (survival rate 90%)	A. Aslam et al. (2014)

#### 2.6.4 Antiparasitic Activity

Coccidiosis is the most economically important poultry parasitic infection in intensive poultry production systems, and drug resistance, together with the strict limitation of the discovery of new coccidocidal/coccidiostat drugs, has forced the search for alternative solutions, including herbal products (Cervantes et al. 2020). Various herbs and plant extracts with antiparasitic activities have been summarized (Eftekhari‐Hasan‐Abad and Ghaniei 2023; El‐Shall et al. 2022; Shahrajabian and Sun 2024). Among the herbal anti‐parasites, green tea (0.4 g/kg of feed) offers a possible replacement for conventional ionophores to control coccidiosis in broiler chickens (Jelveh et al. 2022), and a natural solution formulated from *Yucca schidigera* and *Trigonella foenum‐graecum* (Norponin XO2) is as efficient as a monensin supplementation in managing coccidiosis in poultry (Benarbia et al. 2022). More interestingly, some of the commercially available herbal anticoccidiosis products are effective, like routinely used chemical drugs such as monensin (Ghaniei et al. 2023) and toltrazuril (Shahininejad et al. 2024). Recent studies indicate that natural coccidiostats, including *Artemisia annua*, *Bidens pilosa* and Garlic (*A. sativum* L.), affect different stages of *Eimeria* life cycles (de Almeida et al. 2012; Ahmad et al. 2024; Coroian et al. 2022; Muthamilselvan et al. 2016), and a combination of different medicinal plants has better effects than one plant alone (Eldeeb et al. 2025; Han et al. 2022; Pop et al. 2019). For example, administration of (5m/LDW) a commercial herbal formula containing a combination of medicinal plants for 10 days post‐inoculation, has been effective in experimental chicken coccidiosis infected with 5 × 104 sporulated oocysts of *Eimeria* spp. (*E. acervulina*, *E. maxima* and *E. tenella*) (Pop et al. 2019). However, some of the medicinal plants with anticoccidiosis activities are presented in Table 4. A few commercialized anti‐coccidiosis herbal products, including BioCoccinTM (natural coccidiostat) and Cocci Phyt, have already been introduced in the poultry market.

**TABLE 4:** The medicinal plants are used as anti‐coccidiosis in poultry production.

Medicinal plant	Poultry parasites	Optimum dose	Impacts	Reference
*Camellia sinensis* (Green tea)	*E. acervulina*, *E. maxima*, *E. necatrix*, *Eimeria tenella*	0.4 g/kg of diet	Feed conversion ratio Alternative to ionophores	Jelveh et al. (2022) Jang et al. (2007)
	*E. acervulina*, *E. maxima*, *Eimeria tenella*	10% of (2% strength W/V) In vitro	Inhibit sporulation Destruction of oocyst wall	Molan and Faraj (2015)
*Artemisia annua*	*Eimeria* species *E. acervulina* *E. maxima* *E. tenella*	1% of diet For 0–14 post inoculation 4–5 mg/kgbw per day 3.5 kg/ton of feed	Inhibit the cell wall formation and sporulation in *Eimeria* species Modulated the inflammatory immune response	Muthamilselvan et al. (2016) Sharma et al. (2024) de Almeida et al. (2022) Coroian et al. (2022) Ghafouri et al. (2023)
*Bidens pilosa*	*Eimeria tenella*	0.01% (0.1 g/kg of feed or 100 ppm) in diet	Suppressed sporozoite invasion, and schizonts, and oocyst sporulation in the life cycle	Yang et al. (2019)
*Quercus infectoria* *Rhus chinensis*, *Terminalia Chebula*	*E. acervulina* *E. brunetti* *E. mitis* *E. maxima* *E. necatrix* *E. praecox* *E. tenella*	Ground powder	Reduce Lesion score Reduce oocyst counts per gram (OPG) of faeces	Gokila et al. (2014) Muthamilselvan et al. (2016) Ghafouri et al. (2023)

#### 2.6.5 Antifungal Activity

Among the fungal infections in poultry, aspergillosis is the most dominant and important fungal disease that affects both poultry farms (breeders, broilers) and hatchery industry. Some of the medicinal plants with antifungal activities are presented in Table 5. However, natural antifungi is used in poultry could be summarized as follow.

**TABLE 5:** The medicine plants are used as anti‐aspergillosis in poultry production.

Medicinal plant	In vitro/ In vivo	Mode of action	Dosage	Reference
*Spinacia oleracea* (concentration 600 mg/mL)	In vitro	Inactivation and death of the toxigenic *A. flavus* conidia	1 mL of herbal extract Per 1 mL of 3 × 10^2^CFU of *A. flavus* spores	Njoki et al. (2017)
*Mentha piperita oil Thymus vulgaris* L. oils	In vivo Air/Litter	Fungicidal activity (reducing fungi colonization)	1/500 to 1/250 (aqueous solutions)	Witkowska et al. (2016)

##### 2.6.5.1 Mycoflora of Poultry Feedstuffs

A wider variety of fungal genera were isolated from litter than from feed, and fungal genera, with dominant *Aspergillus*, *Fusarium*, *Mucor* and *Penicillium* that were isolated in feed, with total densities varying from 7 × 10^2^ to 3.2 × 10^5^ cfu/g. To control fungal contamination in the storage of food, *Spinacia oleracea* (at a concentration of 600 mg/mL) results in inactivation and death of the toxigenic *A. flavus* conidia within 10 min (Njoki et al. 2017).

##### 2.6.5.2 Control of Fungal Contamination in Litter of Poultry Farm

The mean total fungal counts in shavings or wood chips from broiler and layer houses ranged between 10^2^ and 10^8^ cfu/g, and fumigation of the litter with aqueous solution of essential oils (*Mentha piperita* or *T. vulgaris* L. oils) every three days could be an effective prevention method against fungal aerosol in broiler houses (Witkowska et al. 2016).

##### 2.6.5.3 Control of Air Mycoflora in Poultry House

In a farm free from aspergillosis, the concentration of *Aspergillus* spp. in the air varies from 10 to 10^4^ cfu/m^3^, and the negative correlation between relative humidity and the number of *Aspergillus* conidia in air may indicate that xerophilic *Aspergillus* conidia more readily discharge in dry conditions than in a humid atmosphere. Fogging (aerosol particles < 50 µm) of broiler houses with aqueous solution (1/500–1/250) of essential oils (*M. piperita* or *T. vulgaris* L. oils) every three days could be an effective prevention method against fungal aerosol in broiler houses (Witkowska et al. 2016).

#### 2.6.6 Potential Immunomodulatory Activity

**FIGURE 3:**
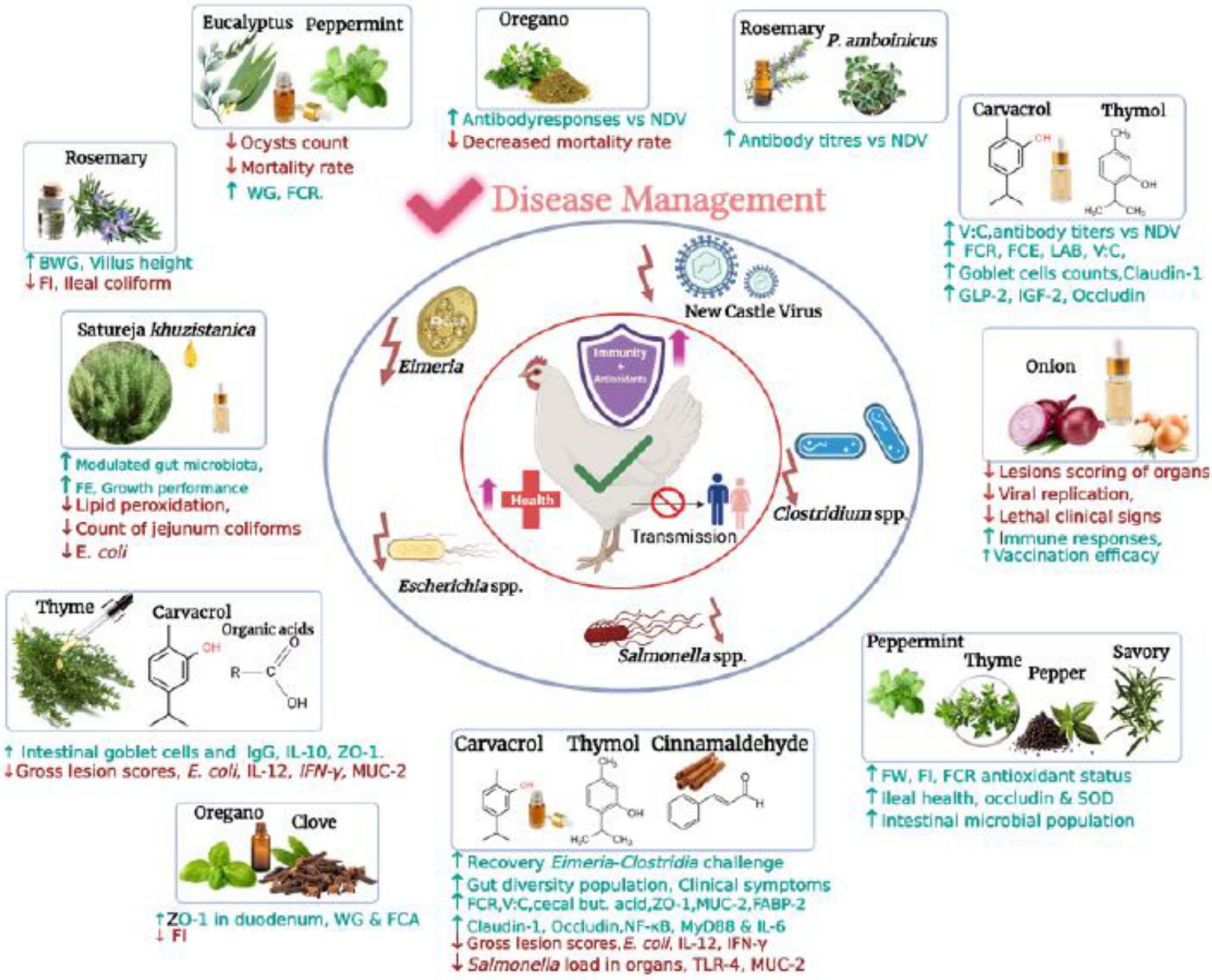
Different EOs and their application alone or in combination for disease management in poultry. Symbol ↑—represents increases or enhancement of investigated parameter. Symbol ↓—represents a reduction, decrease or downregulation of reported measured variable (Toader et al. 2025).

Improvement of resistance by modulation of innate immunity has been suggested as an alternative for antibiotic preventive medication in poultry during the post‐antibiotic era, and some immunomodulator plant‐derived compounds with their mode of action on the innate immune system have been reviewed (Adams et al. 2023). However, some of the medicinal plants' essential oils, such as carvacrol of oregano, are the gold standard essential oils to improve the health of chickens (Meijer et al. 2025), and some of the others with immunomodulatory effects improve immune responses following vaccination in poultry (Hartady et al. 2021; Talebi et al. 2021; Zhai et al. 2011). Oil extract from *N. sativa* is suitable as an adjuvant for AIV vaccines (Mady et al. 2013), and the administration of oil of *N. sativa* following vaccination against AI in poultry improves both humoral and cellular immune responses (Hartady et al. 2021; Talebi et al. 2021). Administration of the extract of *Phyllanthus urinaria* and *Andrographis paniculata* in drinking water improves antibody titer against ND (Astuti et al. 2019). Supplementation of 250 mg/kg of turmeric (*C. longa*) affects the acute phase response (serum amyloid A) following vaccination against ND (Khodadadi et al. 2021), and dietary supplementation of curcumin at a concentration of 100–200 mg/kg significantly increased the production of interleukin‐2 (IL‐2) and interferon‐gamma (IFN‐γ) in broiler chickens (Hafez et al. 2022). A combination of extracts of medicinal plants has also improved humoral immune responses against AI (caused by H9N2 subtype) and IBD (Eladl et al. 2020). It has been suggested that the presence of alkaloids, terpenoids, quinones, simple phenolic compounds, polysaccharides, peptides, glycoproteins and nucleotides of the medicinal plants may be responsible for the immune‐stimulating effect of the herbal products (Chabib et al. 2018). Supplementation of 200 mg/kg nanoprotected REO to the diet of broiler chickens improves IgG and IgM levels (Adil et al. 2024). A diet with 2% leaves powder of green tea (*C. sinensis* L.) + mulberry (*Morus alba*) could improve humoral immune response in broilers (Aziz‐Aliabadi et al. 2023). The latest updates of different EOs and their application for disease management in poultry are presented in Figure 3. Some commercialized immunomodulator herbal products, including Immunofin, Gano‐met and Easy‐immune, have already been introduced in the poultry market.

#### 2.6.7 Potential Activity of Medicinal Plants in Enrichment of Poultry Products

The presence of some bioactive components with antihyperlipidemic activity, such as saponin, tannins and alkaloids, seems to be responsible for the fat‐lowering effect of herbal products in poultry (Saputri and Sumiwi 2020). Dietary supplementation with *A. paniculata* extract or *Zingiber zerumbet* (L.) Smith extract decreased fat and increased protein contents of broiler meats (Rahayu et al. 2019). Inclusion of 350 mg/kg encapsulated FEO (*Foeniculum vulgare* Mill. essential oil) as a phytoestrogen with diets of laying hens improved laying performance by stimulating reproductive hormones in the early and late laying phases (İpçak et al. 2025).

#### 2.6.8 Antioxidant Activity of Herbal Medicine in Poultry

The antioxidant contents of medicinal plants have been reported (Raei et al. 2022; Alberts et al. 2024; Daramola 2019; El‐Sabrout et al. 2023; Kusumawati et al. 2022), and the high content of phenolic compounds in the herbal plants may be involved in the improvement of antioxidant conditions (Li et al. 2017; Sinurat et al. 2018). High amounts of phenolic and flavonoid compounds have stronger antioxidant activity (Alahmadi 2025; Nazari et al. 2023). For example, supplementation of 0.5% rosemary enhanced the activities of serum and liver antioxidant enzymes and increased the expression levels of liver antioxidant genes in broilers (Adil et al. 2024; Christopoulou et al. 2021; Wang et al. 2024).

#### 2.6.9 Anti‐Inflammation Activity

Medicinal plants have anti‐inflammatory effects in both acute and chronic inflammation cases with effects comparable to those of phenylbutazone (Azab et al. 2016; Nunes et al. 2020), and the presence of phytochemicals or bioactive compounds (flavonoids, alkaloids, saponins, phenol, oxalate, etc.) confers the plant the ability to perform anti‐inflammatory activity (Khallaf et al. 2025; Omolere and Alagbe 2020). Some anti‐inflammation herbal products, including Broncofin PLUS, Anzofin and Anzofin Extra, have already been introduced in the poultry market.

#### 2.6.10 Role of Herbal Products in Reducing Greenhouse Gas Production by Poultry

**FIGURE 4:**
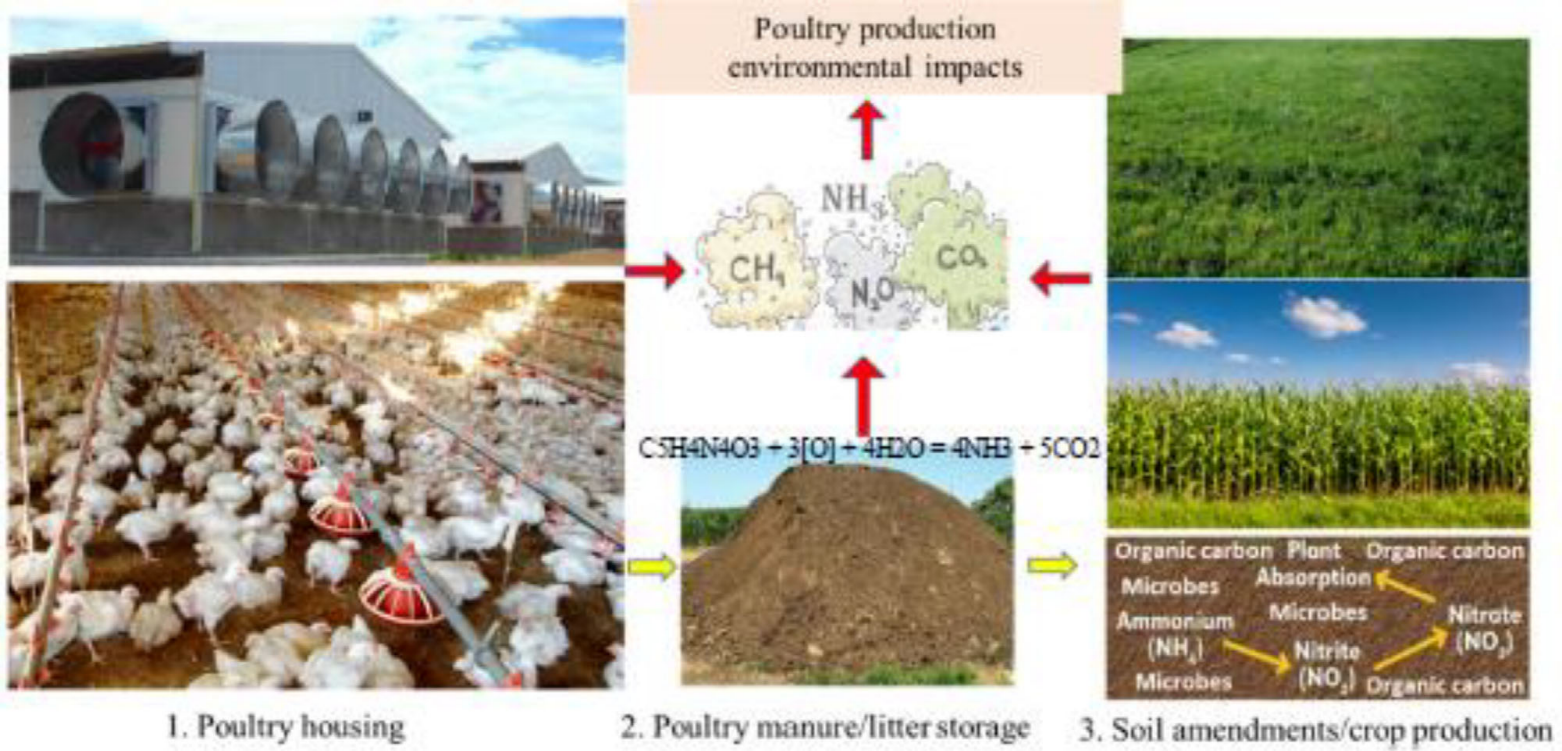
Poultry production impacts on environment (air pollution and greenhouse gas production) (Bist et al. 2023).

Poultry has the lowest carbon footprint of all meat‐producing animals, and the poultry industry is more friendly (g CO2 equivalents/g protein in poultry is 23–37, while in beef it is 194) to the environment than other livestock (Attia et al. 2024; John and Mishra 2024). However, it has been estimated that 8% (606 × 10^6 tons of CO_2_) of the livestock sector emissions of greenhouse gases (GHGs) come from the poultry industry, and the manure is responsible for 6%–20% of the emissions (Attia et al. 2024). Once excreted, nitrogen of urea (4%–12%), uric acid (40%–70%) and nitrogen of feed protein (10%–40%) are quickly hydrolysed to form NH_3_ in the manure as shown in Figure 4 (Bist et al. 2023). The most recommended solutions are the use of phytofeed additives to reduce ammonia emission from poultry farms (Bist et al. 2023; Li, et al. 2023b; Oliveira et al. 2021). For example, application of herbal products such as Yucca (*Y. schidigera*) extract in feed (100–200 mg/kg of diet) and 5 mL/100 L of drinking water has been used to reduce the ammonia content by reducing ammonia levels released by birds (Patoary et al. 2020; Saeed et al. 2018). It has been reported that saponins (widely distributed plant glycosides in *Y. schidigera*) (Kozłowski et al. 2022) of the herbal products affect the GHG emissions by poultry and so far, some commercial products (Alquernat Yucca, BioQuil Gold, Bovaer and anti‐ammonium extra) have been introduced to poultry markets (Elhetawy et al. 2025; Li, et al. 2023c; Press et al. 2000).

#### 2.6.11 Role of Plants in Poultry Vaccine Production

**FIGURE 5:**
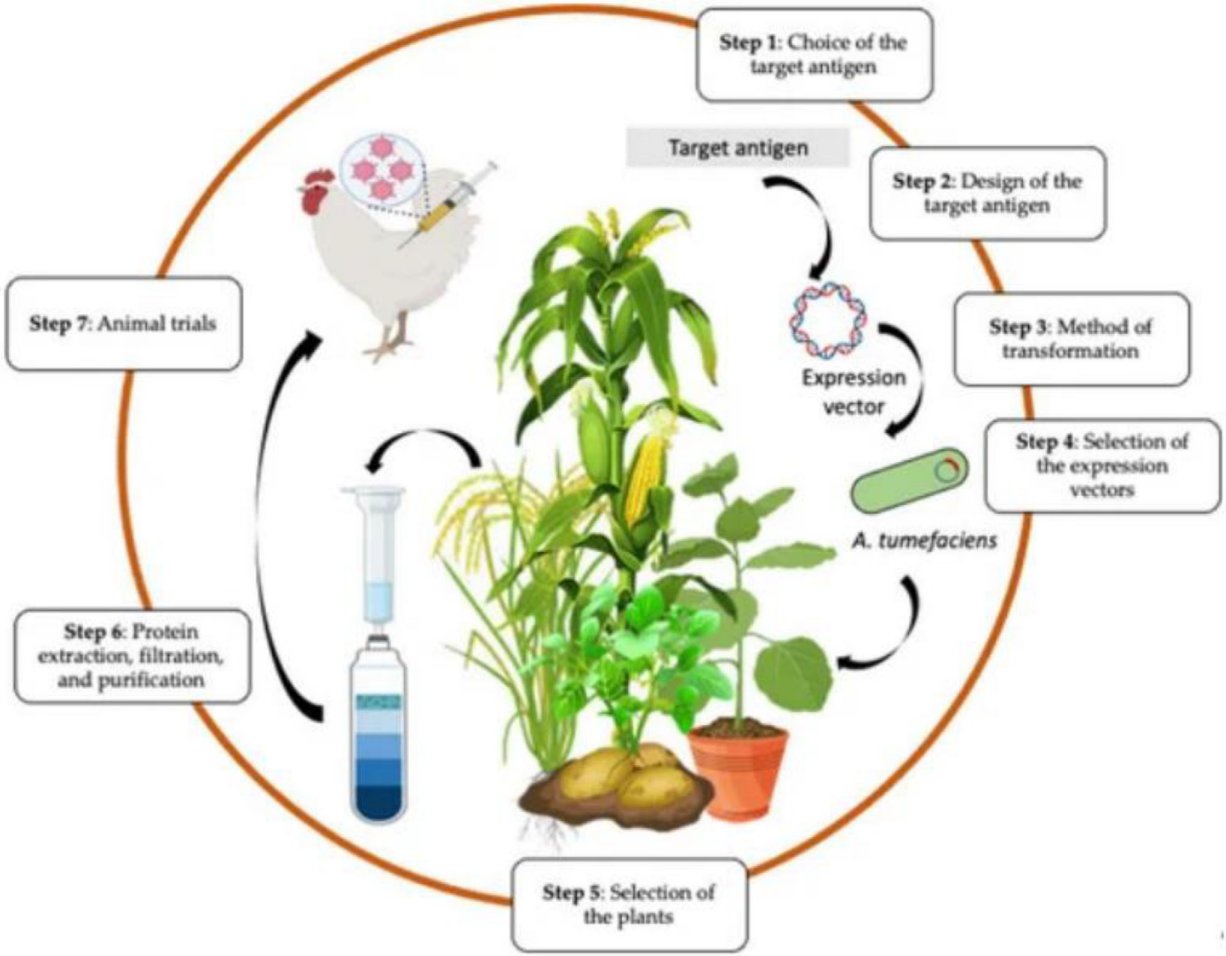
Summary of steps for production of plant‐based vaccines (Nurzijah et al. 2022).

Plant‐based engineering technology (Takeyama et al. 2015) together with genetic engineering has led to transgenic plants being promising ideal alternative platforms to the traditional methods for the production of recombinant plant‐based vaccines in eradication strategies in poultry (Ma et al. 2020). The production of plant‐based virus vaccines is shown in Figure 5, and up to now, the plant‐derived vaccines include both subunit and virus‐like particle (VLP) (Ma et al. 2020; Nurzijah et al. 2022). Some plant‐based vaccines against ND (Guerrero‐Andrade et al. 2006; Ma et al. 2020; Nurzijah et al. 2022; Shahid et al. 2020; Shahriari et al. 2016; Shahriari et al. 2019; Takeyama et al. 2015; Vermij et al. 2006), AI (Nurzijah et al. 2022; Phan et al. 2020), IB (Zhou et al. 2004), IBD (Wu et al. 2007) and *M. gallisepticum* (Mugunthan et al. 2024) have been reviewed. Recently, production of oral route (in feed) of vaccines against NDV (Shahid et al. 2020) has been encouraging, and production of heat‐stable poultry vaccines in the seeds of special plants, including maize, wheat, oat, barley and soybean, will be ideal for their commercial prospects.

#### 2.6.12 Impact of Herbal Medicinal Products on Poultry Blood Profiles

Application of ginger, moringa and garlic reduces abdominal fat content in poultry (Andriyanto et al. 2016; Sugiharto et al. 2018). Supplementation of 200 mg/kg nanoprotected REO to the diets of chickens has a positive influence on blood biochemistry parameters and haematological indices (Adil et al. 2024). Application of 1% green tea leaf powder (GTP) in the diet considerably reduced the low‐density lipoprotein (LDL) and malondialdehyde‐modified (MDA) levels in the serum (Aziz‐Aliabadi et al. 2025). Yucca (*Y. schidigera*) extract is a green feed additive that improves egg quality (egg weight, albumen height and weight and yolk weight) of laying hens (Mao et al. 2023).

## 3 Conclusions

- Promoting health for all and the one‐health system is not what we do; it is who we are.
- During the transit period from the antibiotic era to the post‐antibiotic era, a harmonized global surveillance and detection of antibiotic usage and AMR is recommended.
- Centuries of use in traditional medicine practices can be used as testimony that the medicinal plants have played an essential role in the development of human culture, and their ingredients are effective, safe and have fewer side effects.
- The World Health Organization (WHO) reported that 80% of the Earth's population relies on traditional medicine for their primary health care needs.
- Medicinal plants have the potential to play an essential role in the development of one‐health as a health for humans, animals and the environment.
- Application of medicinal plants in poultry production is the best alternative choice for the post‐antibiotic era.

## 4 Recommendations and Future Directions

The scientists have an obligation to conduct pre‐determined scientific research in‐depth on the medicinal plants to explore the precise medicinal properties of the plants and validate their use (Abreu et al. 2023; Eftekhari‐Hasan‐Abad and Ghaniei 2023; Pashaei et al. 2024) as follows:

The first priority of research is to study in‐depth and to focus on:

- Detection of medicinal plants and encouraging their cultivation.
- Medicinal plants with effects on improving gut‐health and naturally resistant against harmful environments as a phytofeed additive.
- Medicinal plants acting as a general immunomodulator for the innate defence system of birds and following vaccinations against avian pathogens in poultry.
- Medicinal plants with antibacterial, antiviral, antifungal and antiparasitic active ingredients to tackle poultry infections.
- Development of the herbal medicine industry.

The second priority of research is to commercialize traditional medicine (mechanism of action and practices) in modern medicine; therefore, the following items on herbal medicine have to be verified:

- Analyses of active constituent pharmacokinetics and bioavailability in target poultry tissues.
- Elucidation of mechanisms of action and investigation of potential herb–drug/herb–herb interactions.
- Dose‐response trials to optimize MIC50 and MIC90 levels in vivo and in vitro.
- Exploring the best routes of administration in poultry.
- Rigorously controlled long‐term productivity studies.
- Systematic safety and toxicity evaluations in poultry performance (broilers, layers, breeders).
- Studies on residues in poultry products and determination of withdrawal time.
- Shelf‐life impacts on final poultry products.
- Cost‐benefit analysis for commercial formulation.
- Impacts of herbal medicine on the environment, including the soil and water microbiome of the Earth.
- Practical regulations have to be applied to minimize the misuse of herbal medicine.
- As the microbes are intelligent, studies should also focus on encountering microbial resistance to herbal medicine in long‐term administration.

The third priority is to include the well‐defined medicinal plants commercialized products in the education and training of veterinary students' programs.

The fourth priority is to monitor the use of the medicinal plant products regularly to evaluate the progress in phytotherapy and to detect their administration challenges in order to improve evidence‐based practices of herbal medicine in the poultry industry.

The fifth priority is creating awareness among the general population, farmers, veterinarians, healthcare professionals and environmentalists.

Overall, surveillance strategies for monitoring drug residues in poultry products must be implemented regularly to maintain the quality and safety of the products.

## Author Contributions

Initial draft of the MS was written by A. Talebi and A. Savari made a substantial contribution in providing additional reference, significant role in critical revising, English correction and final prof of the MS.

## Funding

The authors have nothing to report.

## Ethics Statement

The authors have nothing to report.

## Conflicts of Interest

The authors declare no conflicts of interest.

## Data Availability

The data that support the findings of this study are available from the corresponding author upon reasonable request.
